# Adapalene-loaded poly(*ε*-caprolactone) microparticles: Physicochemical characterization and *in vitro* penetration by photoacoustic spectroscopy

**DOI:** 10.1371/journal.pone.0213625

**Published:** 2019-03-21

**Authors:** Jessica Mendes Nadal, Guilherme dos Anjos Camargo, Andressa Novatski, William Roger Macenhan, Daniele Toniolo Dias, Fernanda Malaquias Barboza, Amanda Lyra, João Ricardo Roik, Josiane Padilha de Paula, Aloisi Somer, Paulo Vitor Farago

**Affiliations:** 1 Postgraduate Program in Pharmaceutical Sciences, Department of Pharmaceutical Sciences, State University of Ponta Grossa, Ponta Grossa, Paraná, Brazil; 2 Postgraduate Program in Health Sciences, State University of Ponta Grossa, Ponta Grossa, Paraná, Brazil; 3 Department of Physics, State University of Ponta Grossa, Ponta Grossa, Paraná, Brazil; 4 Academic Department of Physics, Federal University of Technology–Paraná, Ponta Grossa, Paraná, Brazil; Northeastern University, UNITED STATES

## Abstract

Adapalene (ADAP) is an important drug widely used in the topical treatment of acne. It is a third-generation retinoid and provides keratolytic, anti-inflammatory, and antiseborrhoic action. However, some topical adverse effects such as erythema, dryness, and scaling have been reported with its commercial formula. In this sense, the microencapsulation of this drug using polyesters can circumvent its topical side effects and can lead to the enhancement of drug delivery into sebaceous glands. The goal of this work was to obtain ADAP-loaded poly(ε-caprolactone) (PCL) microparticles prepared by a simple emulsion/solvent evaporation method. Formulations containing 10 and 20% of ADAP were successfully obtained and characterized by morphological, spectroscopic, and thermal studies. Microparticles presented encapsulation efficiency of ADAP above 98% and showed a smooth surface and spherical shape. Fourier transform infrared spectroscopy (FTIR) results presented no drug-polymer chemical bond, and a differential scanning calorimetry (DSC) technique showed a partial amorphization of the drug. ADAP permeation in the Strat-M membrane for transdermal diffusion testing was evaluated by photoacoustic spectroscopy (PAS) in the spectral region between 225 and 400 nm after 15 min and 3 h from the application of ADAP-loaded PCL formulations. PAS was successfully used for investigating the penetration of polymeric microparticles. In addition, microencapsulation decreased the *in vitro* transmembrane diffusion of ADAP.

## Introduction

**Fig 1:**
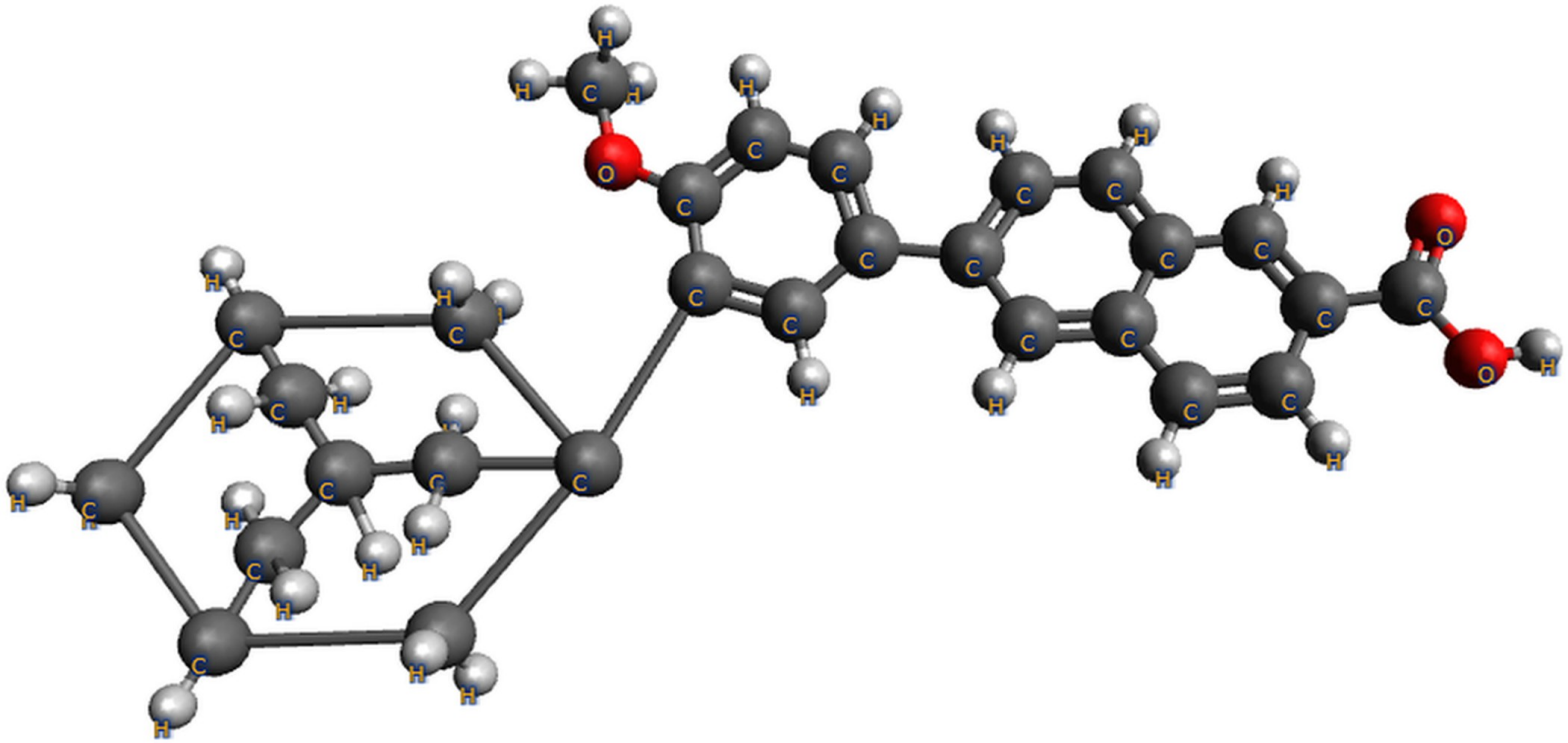
Chemical structure of adapalene (ADAP).

Adapalene (ADAP; C_28_H_28_O_3_; MW: 412.52 g.mol^-1^) or 6-[3-(1-adamantyl)-4-methoxyphenyl]-2-naphthalene-2-carboxylic acid (Fig 1) is a synthetic analog of retinol (vitamin A) used for the acne treatment [1,2]. The mechanism of action of ADAP is due to the selective interaction by specific retinoid intranuclear receptors (RARs), leading to the reduction of sebum production by sebaceous glands, reversing inflammatory lesions, follicular hyperkeratinization and the microcomedones formation [3–6]. The mechanism of action of ADAP also decreases the expression of the toll-like receptors 2 (TLR2) used by the *Propionibacterium acnes*, inhibiting the release of pro-inflammatory cytokines [4,5,7,8]. In addition, the modulation of the immune response occurs by altering the expression of CD1-d and IL10, causing an increase in the antimicrobial activity of the immune system [9–11]. During the early course of ADAP treatment, some local adverse events can be noticed such as: erythema, dryness, peeling, burning, and itching. To minimize these side effects, users need to decrease exposure to sunlight, avoid extreme temperatures, and use moisturizers [12].

In recent years, polymeric microparticles have aroused increasing interest as drug delivery systems. Many authors have reported that microparticles can be used to deliver active compounds into the skin in greater amounts than conventional topical formulations [13]. These microparticles are particularly interesting for the development of controlled release dosage forms. The microparticles also play an important role as drug carriers aiming to improve drug stability and minimize side effects [14]. Various methods are readily available for the microencapsulation of drugs, and one of the most commonly used is the simple emulsion/solvent evaporation [15].

Poly(ε-caprolactone) (PCL) is a linear synthetic polyester that is hydrophobic, biodegradable, and biocompatible. Because of the slow degradation and high stability, this polymer is suitable for the long term and controlled release of encapsulated drugs [16,17]. The PCL undergoes degradation by the hydrolysis of the ester linkages, generating ε-hydroxycaproic acid. However, because of the high hydrophobicity, this reaction is slow and does not produce the acidic environment that occurs when using other polymers such as poly lactic acid (PLA) and poly lactic-*co*-glycolic acid (PLGA) [18]. Besides, the PCL mechanism of degradation includes bulk erosion in the aqueous medium and shows *in vitro* stability, facilitating its use in drug-controlled release systems [19].

One of the greatest current challenges for developing topical formulations is confirming if they permeate through different skin layers. In the case of pharmaceutical products, the active substance should reach the region of interest and maintains its properties even if the skin conditions undergo significant changes regarding moisture content, pH, and oxidative degradation [20]. Photoacoustic spectroscopy (PAS) [21] has been widely used as an alternative method to determine the percutaneous penetration of topically applied substances [20,22–24]. In particular, PAS is a non-destructive spectroscopic method that enables the carrying out of experiments that determine the drug penetration profile along skin thickness at depths up to about 200 μm. Thus, this method can be both used to study the penetration and drug distribution characteristics on the skin [20,22–24]. Although previous papers have widely established the application of PAS for predicting skin drug penetration, no previous report was devoted to investigating its use for evaluating the penetration and drug distribution in synthetic membranes.

Taking into account all these data, the hypothesis of this paper is that the microencapsulation into PCL microparticles may reduce ADAP transmembrane diffusion. At first, the ADAP-loaded microparticles were prepared by emulsion/solvent evaporation and characterized by morphological, spectroscopic, and thermal methods. Then, the encapsulation efficiency was determined by a previously validated UV method. The *in vitro* penetration was performed by photoacoustic spectroscopy using a synthetic membrane (Strat-M) to predict the diffusion through human skin. As far as we are aware, this is the first time that PAS is applied to investigate encapsulated drugs for topical application.

## Materials and methods

### Materials

Adapalene (ADAP, 99.13% pure, Fagron S.A., Rotterdam, Netherlands), poly(*ε*-caprolactone) (PCL) (*M*_*w*_ 10.000 g.mol^–1^, Sigma-Aldrich, St. Louis, USA), poly(vinyl alcohol) (PVA) (*Mw* 72.000 g.mol^–1^, 88.5 mol% of hydrolysis, Vetec, Rio de Janeiro, Brazil), and polysorbate 80 (Tween 80, Delaware, Porto Alegre, Brazil) were used as received. The other reagents and solvents were of analytical grade. Strat-M membrane was purchased from Merck Millipore (Billerica, MA, USA).

### Preparation of adapalene-loaded PCL microparticles

The emulsion/solvent evaporation method was used to prepare the microparticles containing ADAP [16]. The composition of three formulations are presented in Table 1 and was dependent on the amount of ADAP (0, 10, and 20%). First, the ADAP and PCL were dissolved in dichloromethane, forming the organic phase that was added into the aqueous phase composed by poly (vinyl alcohol) (PVA) and polysorbate 80 under mechanical stirring (5000 rev.min^–1^, RW 20 digital overhead paddle stirrer, IKA Works, Wilmington, NC, USA) for 5 min. The emulsion was kept under mechanical stirring (800 rev.min^–1^, RW 20 digital overhead paddle stirrer, IKA Works, Wilmington, NC, USA) at room temperature for solvent evaporation. After 4 h, microparticles were separated by centrifugation (2000 rev.min^–1^, 5 min, BE-4004 centrifuge, Bio Eng, São Paulo, Brazil) and washed three times with purified water to remove the adhered PVA on the surface of particles and the organic solvent. All samples were dried under vacuum at 35 ± 1°C (TE-395 vacuum oven, Tecnal, Piracicaba, Brazil) for 6 h by sifting the powder through mesh 20 after drying. The obtained microparticles were stored in a desiccator under vacuum at room temperature. All formulations were obtained in triplicate. The negative control was the unloaded-microparticles (F0). A physical mixture (PM) containing ADAP and PCL at 1:1 ratio was also made.

**Table 1:** Composition of adapalene-loaded PCL microparticles.

*Composition*	*Formulation*		
	F0 (0%)	F10 (10%)	F20 (20%)
**Aqueous phase**			
Polysorbate 80 (g)	0.50	0.50	0.50
PVA (g)	4.00	4.00	4.00
Purified water (mL)	200.0	200.0	200.0
**Organic phase**			
Adapalene (ADAP) (g)	—	0.20	0.40
PCL (g)	2.00	1.80	1.60
Methylene chloride (mL)	40.0	40.0	40.0

### Residual moisture

An infrared moisture analyzer (Top 160 Ray, Bel engineering, Monza, Italy) was used to evaluate the water content of pure ADAP, PCL, and microparticles. An amount of 0.050 g of each sample was placed on an aluminum plate and dried at 105°C until a constant weight was reached. The moisture content was obtained from the percentage corresponding to the mass loss. The tests were carried out in triplicate.

### Characterization of adapalene-loaded PCL microparticles

#### Drug loading and encapsulation efficiency

In a volumetric flask, 10 mg of loaded and unloaded-microparticles was dissolved in acetone (20 mL) and stirred at 1000 rpm for 24 h. The volume was filled to 100 mL with purified water and filtered through a poly(vinylidene fluoride) membrane filter (Durapore membrane, 0.22 μm pore size, Millipore, Bedford, USA). After suitable dilution in ethanol, the drug concentration was spectrophotometrically determined at 321 nm (Hewlett Packard 8452A UV-Vis diode array spectrometer, Boeblingen, Germany) in triplicate. The UV method for ADAP quantification was previously validated in terms of linearity, precision, reproducibility, accuracy, and specificity. The concentration range varied from 4 to 12 μg.mL^–1^. Linearity was 0.9998, and the detection limit was 0.35561 μg.mL^–1^. The accuracy was 93.66% for 1.75 μg.mL^–1^, 97.92% for 2.75 μg.mL^–1^, and 97.34% for 3.75 μg.mL^–1^. The repeatability presented a relative standard deviation (RSD) = 3.20, and the intermediate precision showed an RSD = 1.86. The encapsulation efficiency (EE) was then calculated using Eq 1:

$$EE=\left(\frac{mass\;of\;ADAP\;in\;microparticles}{theoretical\;mass\;of\;ADAP}\right)x100$$ (1)

#### Field emission gun scanning electron microscopy (FEG-SEM)

The microparticles (F0, F10, and F20) were mounted on polished aluminum stubs, sputtered with gold (IC-50 Ion Coater, Shimadzu, Kyoto, Japan), and analyzed using a field emission gun scanning electron microscope (FEG-SEM, TESCAN, model MIRA 3, Brno, Czech Republic) at an accelerating voltage of 15 kV with different magnifications.

#### Particle size

The particle size distribution was estimated from the measurement of 200 particles, assuming a spherical shape, observed in an arbitrarily chosen area of enlarged micrographs using the measurement toolbox for MATLAB (Mathworks Inc., Natick, MA, USA). The span was calculated using Eq 2:

$$span=\frac{d_{\left(v,90\right)}-d_{\left(v,10\right)}}{d_{\left(v,50\right)}}$$ (2)

where *d*_(*v*,10)_, *d*_(*v*,50)_, and *d*_(*v*,90)_ are the particle diameters determined, respectively, at the 10^th^, 50^th^, and 90^th^ percentiles of the undersized particle distribution curve.

#### Fourier-transformed infrared (FTIR) spectroscopy

The Fourier-transformed infrared (FTIR) spectra of ADAP, PCL, polymeric microparticles, and PM were recorded on a Shimadzu IR Prestige-21 spectrophotometer. (Kyoto, Japan). 4 mg of each sample were mixed with 100 mg of KBr, and the pellets were prepared under a pressure gauge of 80 kN. The spectra were obtained from 4000 to 400 cm^–1^, with 32 scans and a resolution of 4 cm^–1^ in absorbance mode.

#### Differential scanning calorimetry (DSC)

DSC measurements were performed using a DSC-60 (Shimadzu, Kyoto, Japan). The samples of ADAP, PCL, PM, and microparticles were packed in an Al_2_O_3_ crucible. The heating rate was 10 K.min^−1^ and under dynamic N_2_ atmosphere (flow rate: 50 mL.min^−1^) with a temperature range from 20 to 500°C. The DSC cell was calibrated with indium (m.p. = 429.6 K; ΔHfusion = 28.54 J. g^−1^) and zinc (m.p. = 692.6 K).

### Characterization of Strat-M membrane

#### Photoacoustic analysis

The photoacoustic spectroscopy (PAS) can evaluate the permeation profile by varying the modulation frequency and analyzing the absorption spectra by the photoacoustic (PA) signal. The permeation profile is measured when the drug characteristic band is observed [20,23,24]. The sample depth contributing to the PA signal is estimated using the thermal diffusion length *μ_s_* = (*α/πf*)^1/2^, in which *α* is the sample thermal diffusivity and *f* is the light modulation frequency [20,23,24].

The open photoacoustic cell (OPC) technique was applied as described in [25,26] to obtain the thermal diffusivity of the membrane. The PA signal in a function of *f* for a monochromatic been was recorded and analyzed by Eq 3:

PA=1fC1αs1/2sinh(lsσs)+1f32C2αs3/2sinh(σsls)[cosh(σsls)−σsls2sinh(σsls)−1] (3)

where $\sigma_{s}=(1+i)\sqrt{\pi \;f/\alpha_{s}}$ is the complex diffusion coefficient and C_1_ and C_2_ are adjust constants [25,26]. Five measurements were performed at different points of the membrane.

#### Field emission gun scanning electron microscopy (FEG-SEM) for the membranes

To correlate the thermal diffusivity data and the depth of penetration, the cross section of Strat M was evaluated using the same FEG-SEM applied for microparticles, with 200X of magnification.

#### Photoacoustic spectroscopy of Strat-M membrane

**Fig 2:**
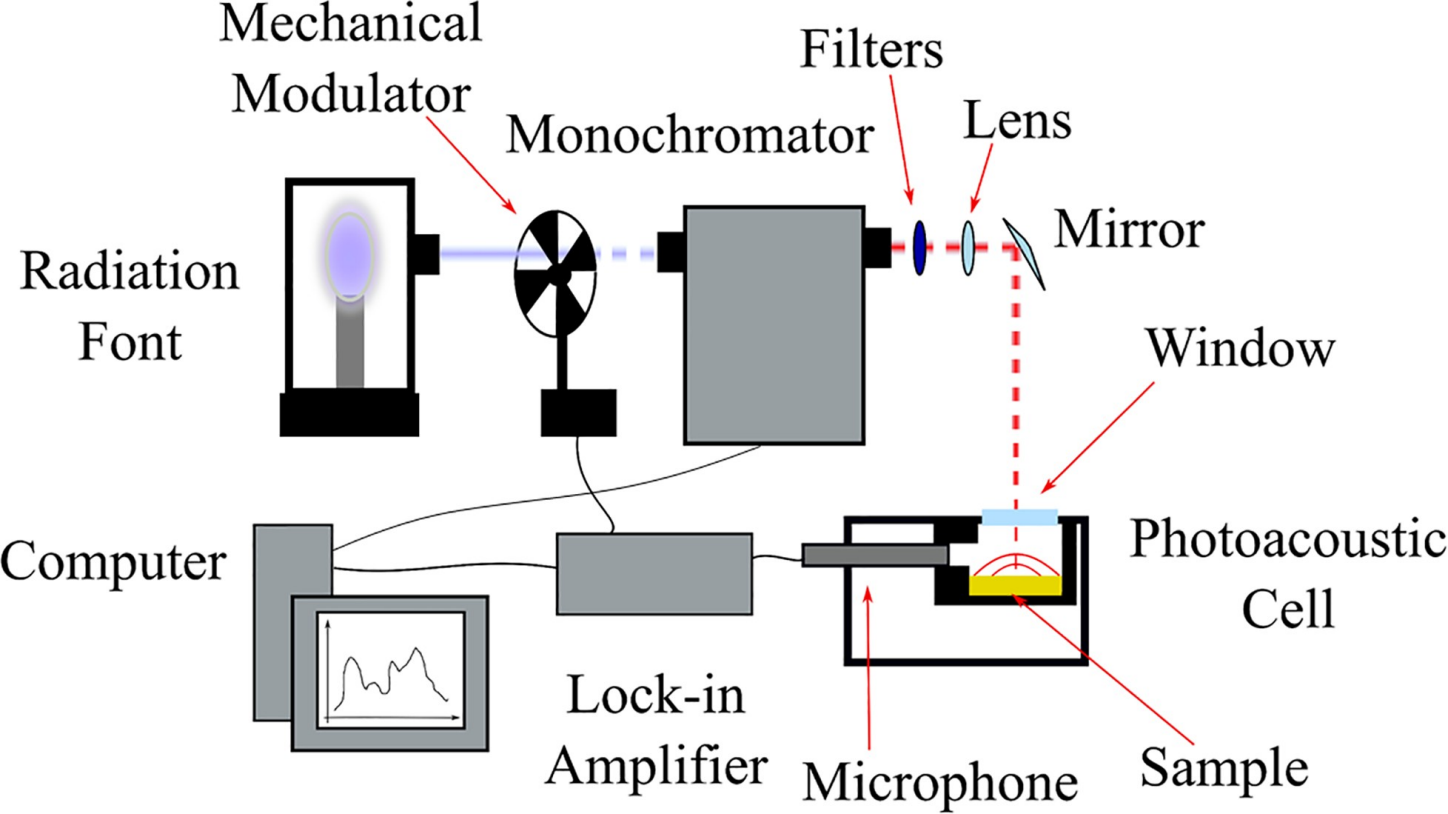
Schematic setup of photoacoustic spectroscopy. The radiation font, the mechanical modulator, monochromator, filters, lens, microphone as the detector, and a personal computer for data acquisition.

The photoacoustic spectroscopy (PAS) homemade setup was used for evaluating the Strat-M membrane, and the schematic apparatus is presented in Fig 2. This apparatus consisted of a Xenon lamp with 800 W (6269, Oriel Instruments, Newport Corporation, Franklin, MA, USA) as the light source, monochromator (CornerstoneTM 260 1/4m, Oriel Instruments), mechanical chopper (SR540, Stanford Research Systems, Sunnyvale, CA, USA), lock-in amplifier (SR830, Stanford Research Systems), and microphone (4953, Brüel and Kjaer Instruments, Santo Amaro, Brazil) coupled to a sealed photoacoustic cell for signal detection. Higher order diffractions were eliminated by bandpass filters. The spectral range for UV-Vis was between 225 and 400 nm because of the ADAP and loaded microparticles characteristics absorptions. The chopper was tuned to different frequencies to modulate the light that impinges the sample. The data acquisition was performed by a personal computer. To correct the source emission intensity in each wavelength, all spectra were normalized with respect to a carbon black sample signal.

### *In vitro* permeation studies

The measurements of the permeation profile were performed using a Strat-M membrane that was lighted on the opposite side of the surface applied with the formulations using the PAS setup. The membrane samples were analyzed after 15 min and 3 h from the application of ADAP, PM, F10, and F20 formulations. To choose the modulation frequencies, two factors were considered: values that could provide a depth profile through the membrane and values that were not multiples of the electrical power grid (60 Hz), which can cause noise effects. Besides, from the OPC technique and FEG-SEM results it was possible to determinate that frequencies 5, 23, 51, and 203 Hz provided satisfactory depth scan through the membrane.

#### Gaussian curve fitting

The absorption spectrum can be determined by a Gaussian curve based on wavelength (λ) because absorption intensity is directly related to the square of dynamic momentum. Thus, the amount of absorbing material is associated with peak area, which can be measured by a multi-peak Gaussian fit. Therefore, Gaussian curves were used in this work to measure peak areas and not to interpret the related absorbing substances. Since the peak positions from the spectra of both formulations and membrane used in the analysis were comparative, we assumed that this mathematical procedure is suitable to quantify the spectra and interpret the permeation of ADAP-loaded microparticles in the synthetic membrane. A detailed report about these analyses is in the supplementary material.

## Results and discussion

### Water content and encapsulation efficiency

After drying, all the obtained formulations showed powder aspect and white color similar to PCL. This result showed that the emulsion/solvent evaporation method successfully achieved the ADAP-loaded PCL microparticles preparation. Water contents of 5.70 ± 0.10% and 1.30 ± 0.10% were obtained for pure ADAP and PCL, respectively. In contrast, microparticles showed only residual moisture values, as presented in Table 2.

**Table 2:** Water content^1^, adapalene-loaded^1^, encapsulation efficiency (EE)^2^, particle size^1^ and span for PCL microparticles.

Microparticles	Water content (%)	Adapalene-loaded (mg.g^–1^)	EE (%)	Mean diameter (μm)	*Span*
F0	3.06 ± 0.09	—	—	8 ± 4	0.89
F10	2.7 ± 0.1	100 ± 2	100.44	8 ± 3	1.25
F20	2.91 ± 0.04	199 ± 2	99.37	7 ± 3	1.02

^1^means (*n* = 3) ± standard deviation

^2^means (*n* = 3)

These data demonstrate that the performed vacuum drying was able to remove the water used during microencapsulation. The residual water content in a powder influences its physical stability and controls the magnitude of capillary forces that hold particles in aggregate [27]. Considering the obtained results, it is possible to expect that the water content has a low interference on the physical stability of PCL microparticles.

Table 2 also summarized the drug content and EE for ADAP-loaded microparticles. High percentages of drug entrapment were obtained for PCL microparticles by simple emulsion/solvent evaporation. Also, all formulations showed suitable EE values higher than 99%. These values are mainly based on the poor aqueous solubility (4.01 μg.L^−1^) of ADAP [28], leading to an increase in the drug loaded into microparticles.

#### Field emission gun scanning electron microscopy (FEG-SEM)

**Fig 3:**
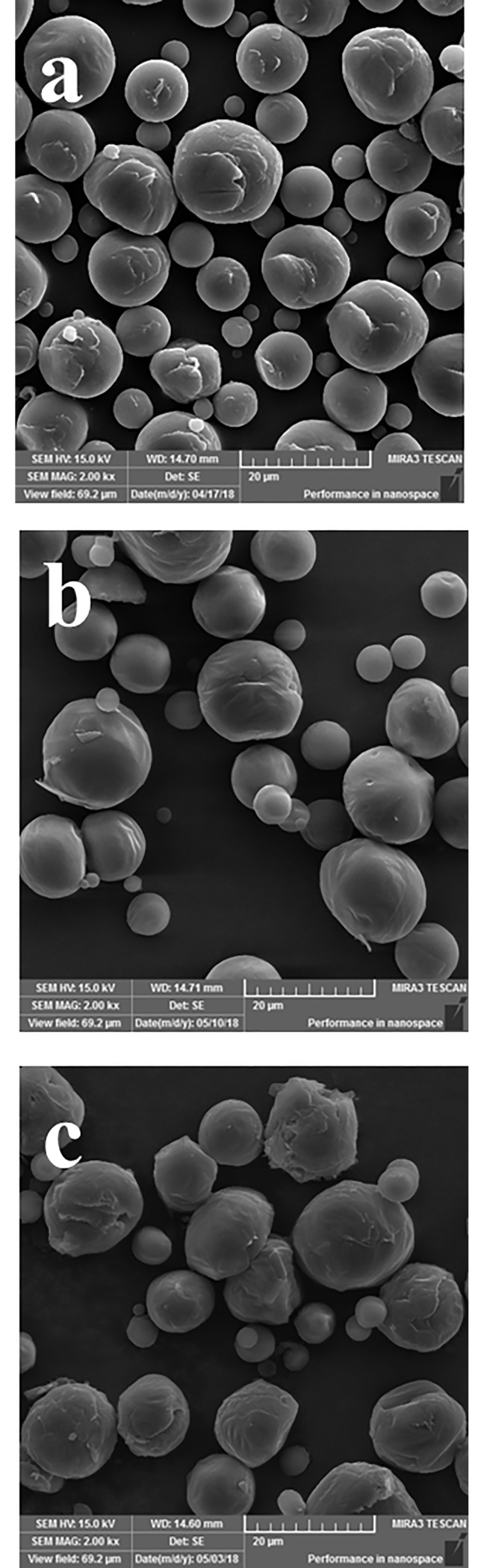
**Scanning electron micrographs of PCL microparticles:** F0 (a), F10 (b) and F20 (c). Magnifications of 2000X presenting a spherical shape and smooth, slightly flaky surface.

FEG-SEM analysis showed that PCL microparticles do not contain pores on their surface. All samples presented a spherical and almost spherical shape and had a smooth and slightly flaky surface (Fig 3). Previous studies that obtained PCL microparticles by the simple emulsion/solvent evaporation technique observed the same morphological data [16,29,30]. Unloaded-microparticles had similar morphology and size to formulations F10 and F20.

#### Particle size

The mean particle sizes obtained for unloaded and ADAP-loaded PCL microparticles are presented in Table 2. All formulations were micrometer-sized. Formulations F0, F10, and F20 showed mean diameters of about 8 *μ*m. Earlier studies demonstrated that microparticles with a diameter ranging from 3 to 10 *μ*m can be randomly distributed into the hair follicles and *stratum corneum* [13,31]. Hence, the particle size obtained from ADAP-loaded PCL microparticles are suitable to site-specific drug delivery to the hair follicles. In addition, PCL microparticles (F0, F10, and F20) revealed span values lower than 2 [32], representing a narrow polydispersity around the mean. Thus, these microparticles had a unimodal behavior, as requested for this topical pharmaceutical form.

### Fourier-transformed infrared (FTIR) spectroscopy

**Fig 4:**
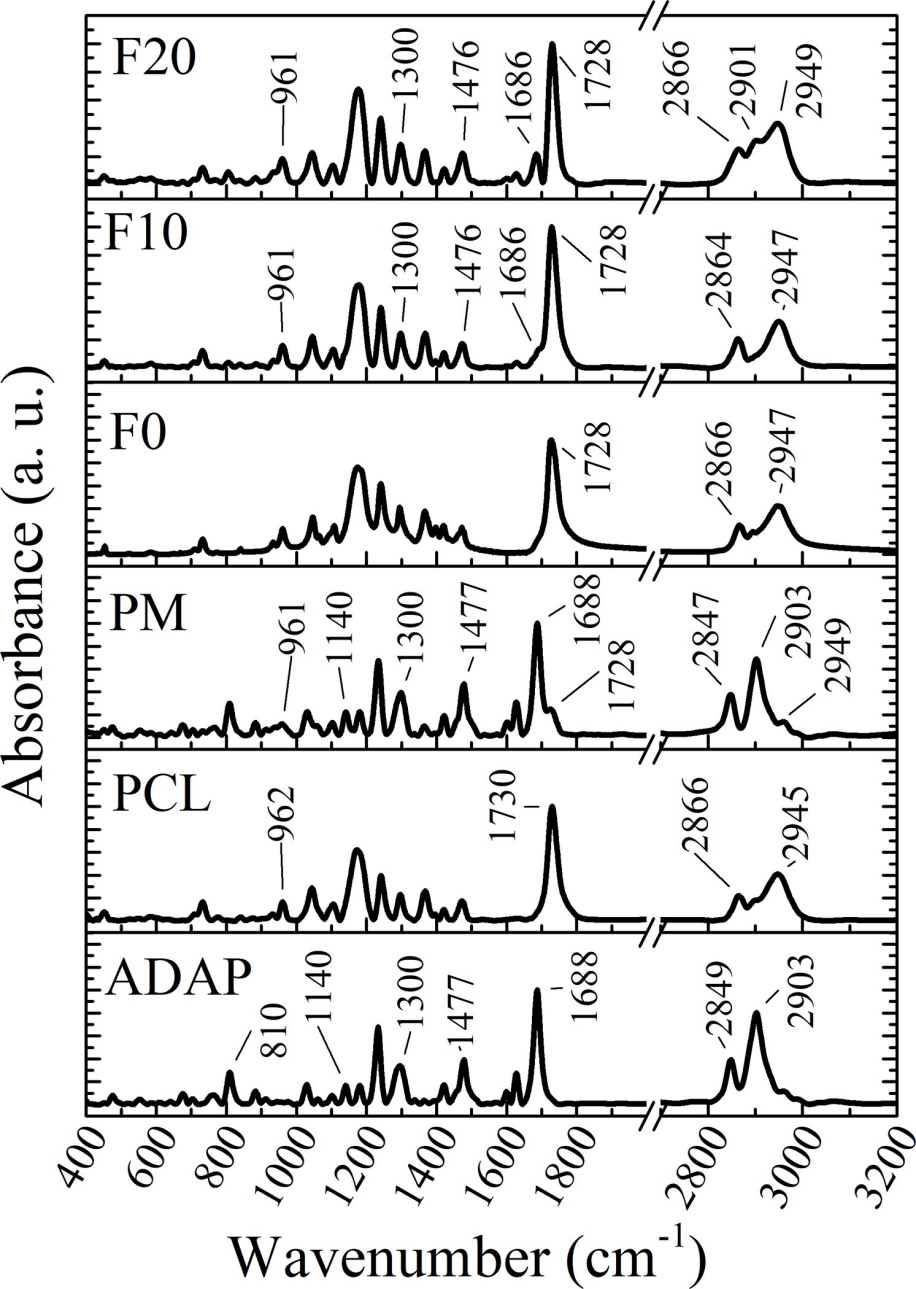
FTIR results of ADAP, PCL, PM, and PCL microparticles (F0, F10, and F20). PM presented bands at 1140, 1300, 1477, 1688, 2847, and 2903 cm^-1^ that are related to ADAP; bands at 961, 1728 and 2949 cm^-1^ that are attributed to PCL. The F10 and F20 do not present any shifts on these bands. In this case, the encapsulation process does not induce chemical interaction between the drug and PCL.

FTIR spectroscopy was performed in order to explore whether the microencapsulation resulted in any chemical change by comparing differences in band assignments among raw materials and formulations. FTIR results of ADAP, PCL, PM, and PCL microparticles are presented in Fig 4. The ADAP infrared spectra revealed absorption bands at 2903 cm^-1^ (C–H stretch in aldehyde and ketone), 2849 cm^-1^ (carboxylic O–H stretch), 1688 cm^-1^ (C = O stretch), 1477 cm^-1^ (C = C stretch), 1300 cm^-1^ (–C–O stretch), 1140 cm^-1^, and 810 cm^-1^ (aromatic stretch, out-of-plane bend) [27,33–35]. The PCL infrared spectrum presented typical bands at 2945 cm^-1^ (asym CH_2_ stretch), 2866 cm^-1^ (sym CH_2_ stretch), 1730 cm^-1^ (ester C = O stretch), and 1060–1150 cm^-1^ (asym C–O–C stretch) [36]. Considering their FTIR spectra, ADAP-loaded PCL microparticles and PM presented band assignments at the same wavenumber ranges as those of the PCL and ADAP results. The broadening peaks at 2866, 2901, and 2949 cm^-1^ for F20 formulation is attributed to O–H and CH_2_ stretch vibrations from the drug and polymer, respectively. Consequently, the microencapsulation process does not induce chemical interactions between the drug and PCL and ADAP, ensuring the drug’s expected anti-acne effect.

### Differential scanning calorimetry (DSC)

**Fig 5:**
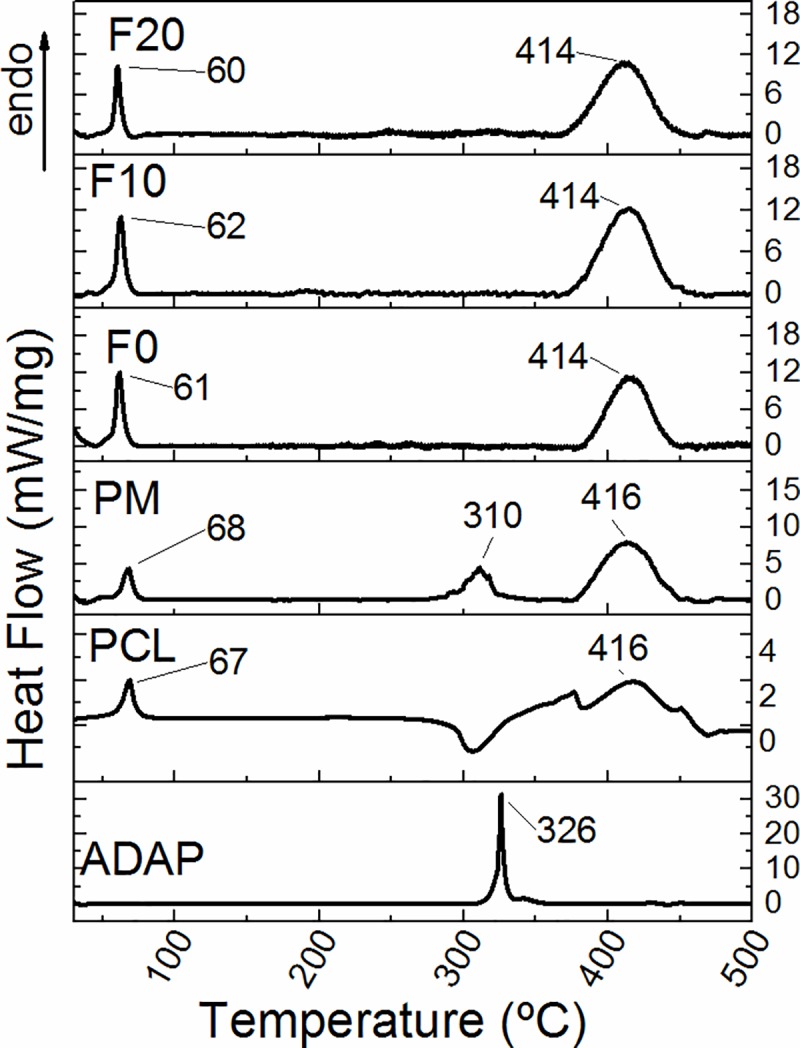
DSC curves of ADAP, PCL, PM, and PCL microparticles (F0, F10, and F20). The formulations F10 and F20 do not present the melting point of ADAP at 326°C, showing a complete drug amorphization.

The DSC results for pure ADAP, PCL, PM, and PCL microparticles are presented in Fig 5. ADAP showed an endothermic event at 326°C related to the melting point in accordance with the literature [35]. PCL presented a melting temperature of 67°C and a decomposition temperature of 416°C (endothermic events), confirmed by previously reported data [36]. The endothermic event at 305°C can be related to a first event degradation process due to the rupture of the polyester chains of PCL [37]. PM sample presented endothermic peaks at 68 and 416°C attributed to melting point and decomposition of PCL, respectively. The peak at 310°C is because of the melting point of ADAP. For unloaded and ADAP-loaded microparticles (F0, F10, and F20), we noticed the endothermic events at 61 and 414°C that attributed to the melting and decomposition points of PCL. There are no events attributed to ADAP in these samples, indicating a complete drug amorphization. The lack of events is probably because of the loosening crystal forces of ADAP molecularly dispersed within PCL that can favor an increase of drug water solubility [38].

### Strat-M membrane characterization

**Fig 6:**
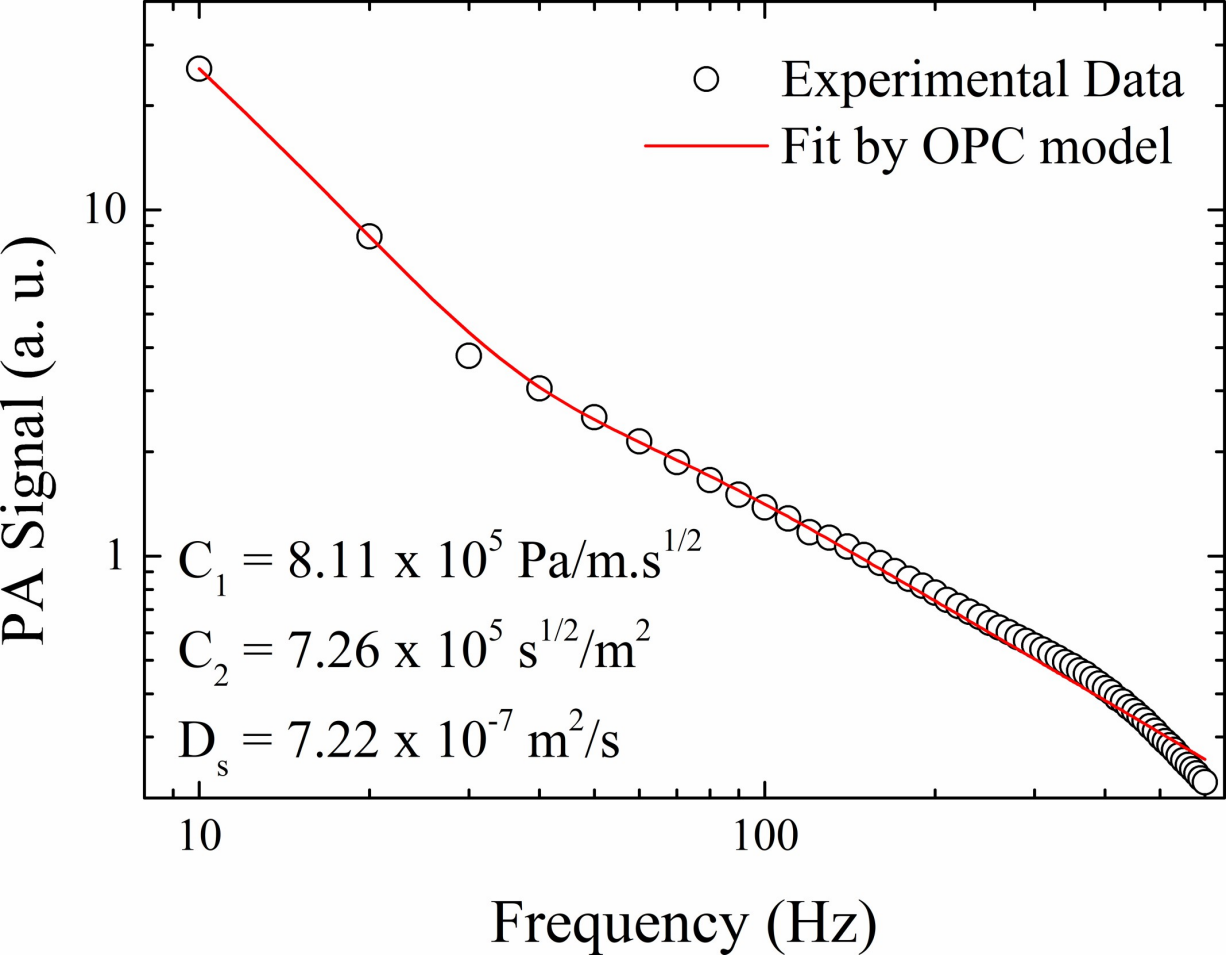
Experimental data of frequency scan by OPC technique for the Strat-M membrane with a thickness of 310±10 μm. The solid line represents the best data fit by Eq (2).

To obtain the depth profile through the membrane, it is necessary to determinate the thermal diffusion length (μ) that measures the thickness of the parcel of the sample that most contributes to PA signal generation. In the present permeation study, this category of thermal thickness is proportional to the thermal diffusivity of the Strat-M membrane which is unknown. In this context, Fig 6 shows a frequency scan of the PA signal obtained by the OPC technique for the Strat-M membrane with a thickness of 310 μm. A good agreement of experimental data and theoretical fit by Eq (2) was observed, and the mean thermal diffusivity measured was *α_s_* = (7.0±0.5)×10^−7^ m^2^.s^-1^. This value has the same order of magnitude as those of human skin (~ 1.36 x 10^−7^ m^2^.s^-1^) and of synthetic polymeric materials (~1.1 x10^-7^ m^2^.s^-1^, which is typical for low-density polyethylene and polyethylene terephthalate) [36]. As far as we are aware, this is the first report determining the thermal diffusivity for the Strat-M membrane. Therefore, modulation frequencies at 5, 23, 51, and 203 Hz will provide a depth scan (μ_membrane_) of 210, 100, 67, and 34 μm, respectively.

**Fig 7:**
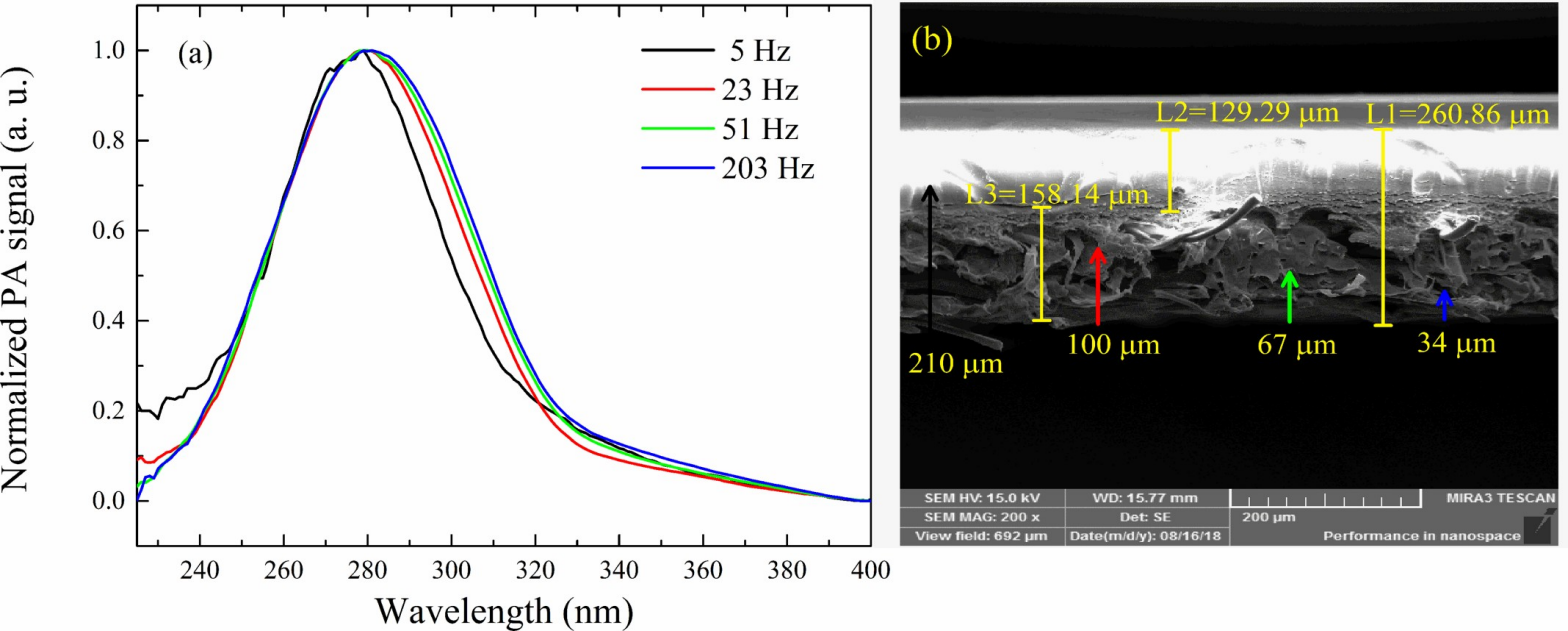
(a) PAS result for the Strat-M membrane. We can notice a redshift with decreasing depth, indicating the difference of the polymeric layers from the membrane. (b) FEG-SEM image of the membrane with 200x of magnification. The arrows illustrate the thermal diffusion length (μ_membrane_) obtained for frequencies of 5, 23, 51, and 203 Hz, and the colors are the same as the respective spectrum from (a).

The spectra obtained by PAS for the Strat-M membrane at 5, 23, 51, and 203 Hz are presented in Fig 7A. We can notice a redshift by increasing the modulation frequency, and the shift becomes more prominent from 5 to 23 Hz (210 to 100 μm in depth). This shift is attributed to the difference between the polymeric layers of membrane, which is explored in the following paragraphs. FEG-SEM results (Fig 7) confirmed the difference between the layers. For better visualization, the depth lengths (μ_membrane_) are marked with arrows.

According to the literature [32,34], the Strat-M membrane is composed of two layers of poly(ether sulfone) with a very tightly packed surface that provides some opposition to drug permeation. Above these layers, a layer of polyolefin is placed with a more open net that is therefore more permeable. From these polymeric layers, a porous structure with blends of synthetic lipids is provided that furnishes a gradient of pore size that changes diffusivity. The difference in pore sizes is shown in Fig 7B.

To further explore the PAS results for the Strat-M, a fit using two Gaussian curves for all spectra was performed that had a peak centered at 280 nm and a broad band centered at 300 nm. The band at 280 nm remains at the same position for the spectra for all frequencies (see supplementary material). In contrast, the broad band maintained the half-width of 100 nm, but the center shifted from 300 nm at 5 Hz to 350 nm at 203 Hz. These bands are related to the π→π⃰ transition associated with the π electrons in the benzene rings and mostly a function of the intra-chain interaction [39,40]. The shift from 300 to 350 nm means the polymeric chains become less entangled, so for a modulation frequency of 5 Hz (210 μm), the generated photoacoustic signal was from the poly(ether sulfone). The other frequencies generated photoacoustic signals in the polyolefin layer. Summarizing, the spectroscopic behavior of membrane with different values of modulation frequencies is associated with the change in the membrane layer. This is an important step in understanding the structure of Strat-M membrane, which is necessary to understanding *in vitro* permeation studies.

### *In vitro* permeation study

**Fig 8:**
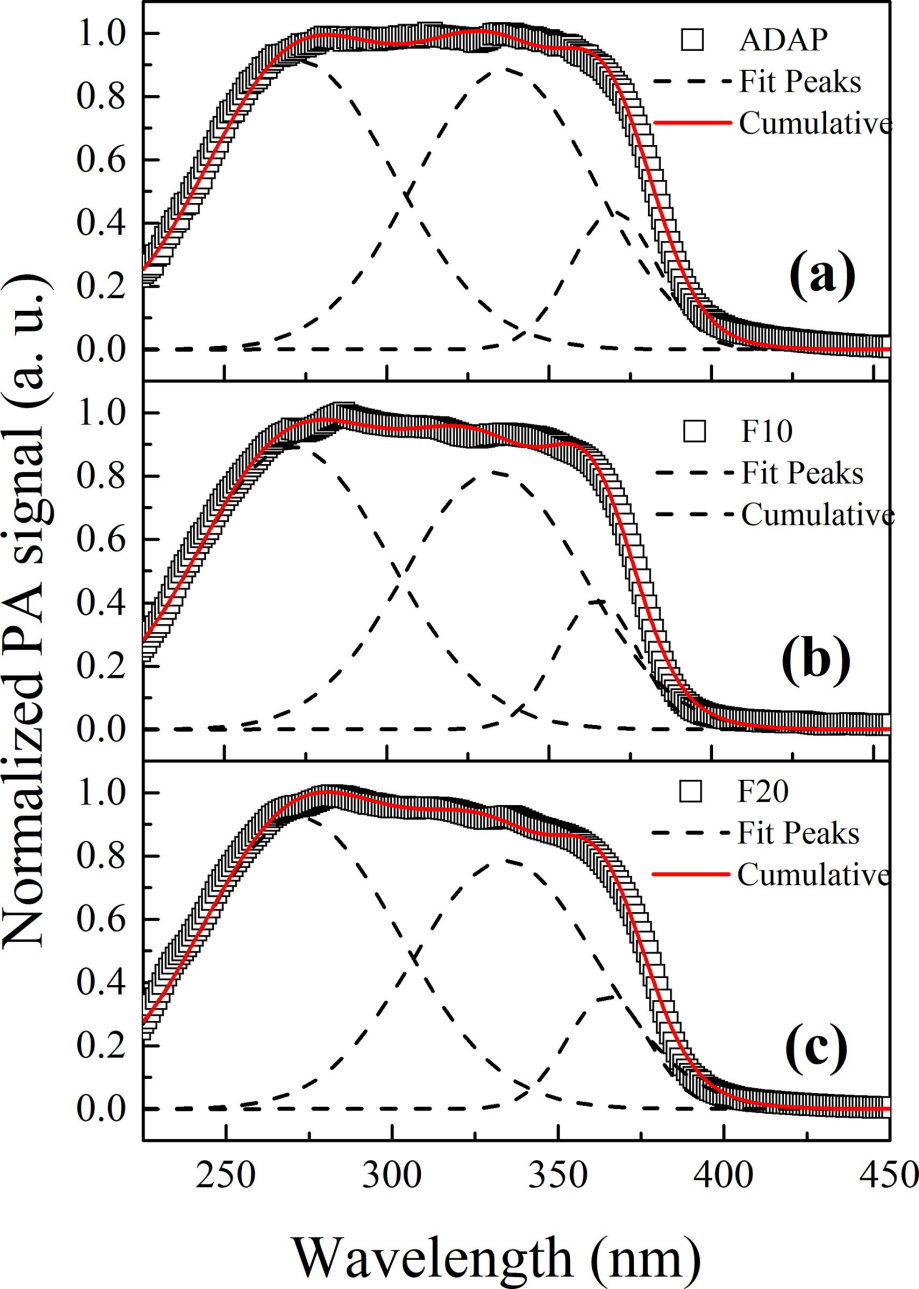
Photoacoustic spectra of formulations. ADAP and ADAP-loaded PCL microparticles. The Gaussian curves are centered at 272, 336, and 369 nm. The bands at 336 and 369 nm decrease in intensity for F10 and F20, indicating the enclosure of the drug molecules into polymeric microparticles.

To determine the permeation of ADAP in Strat-M membrane, the absorption spectra of ADAP and formulations of ADAP-loaded PCL microparticles must first be obtained. Fig 8A shows the ADAP spectra obtained by PAS spectroscopy, in which three Gaussian curves centered at 272, 336, and 369 nm were used to obtain the total spectrum (see supplementary material) in accordance with the literature [41,42]. F10 and F20 formulations (Fig 8B and 8C) were fitted with the Gaussian curves using the same position and width parameters of the ADAP. We can notice a decrease in intensity of the bands centered at 336 and 369 nm. These bands are attributed to transitions between π-electron energy levels of the aromatic rings of ADAP ligand. This decrease is an indication that the drug molecules are enclosed in polymeric microparticles.

To detect the formulation after application on the synthetic membrane, the redshift of the broad band of Strat-M with increasing modulation frequency was considered. The total spectrum was constructed by simulating each contribution using the absorption bands widths and centers of the formulations (Fig 8) and untreated membrane (Fig 7). The intensities of Strat-M bands were maintained constant. Because PCL did not show a PA signal, we considered the ADAP Gaussians curves for the PM sample.

**Fig 9:**
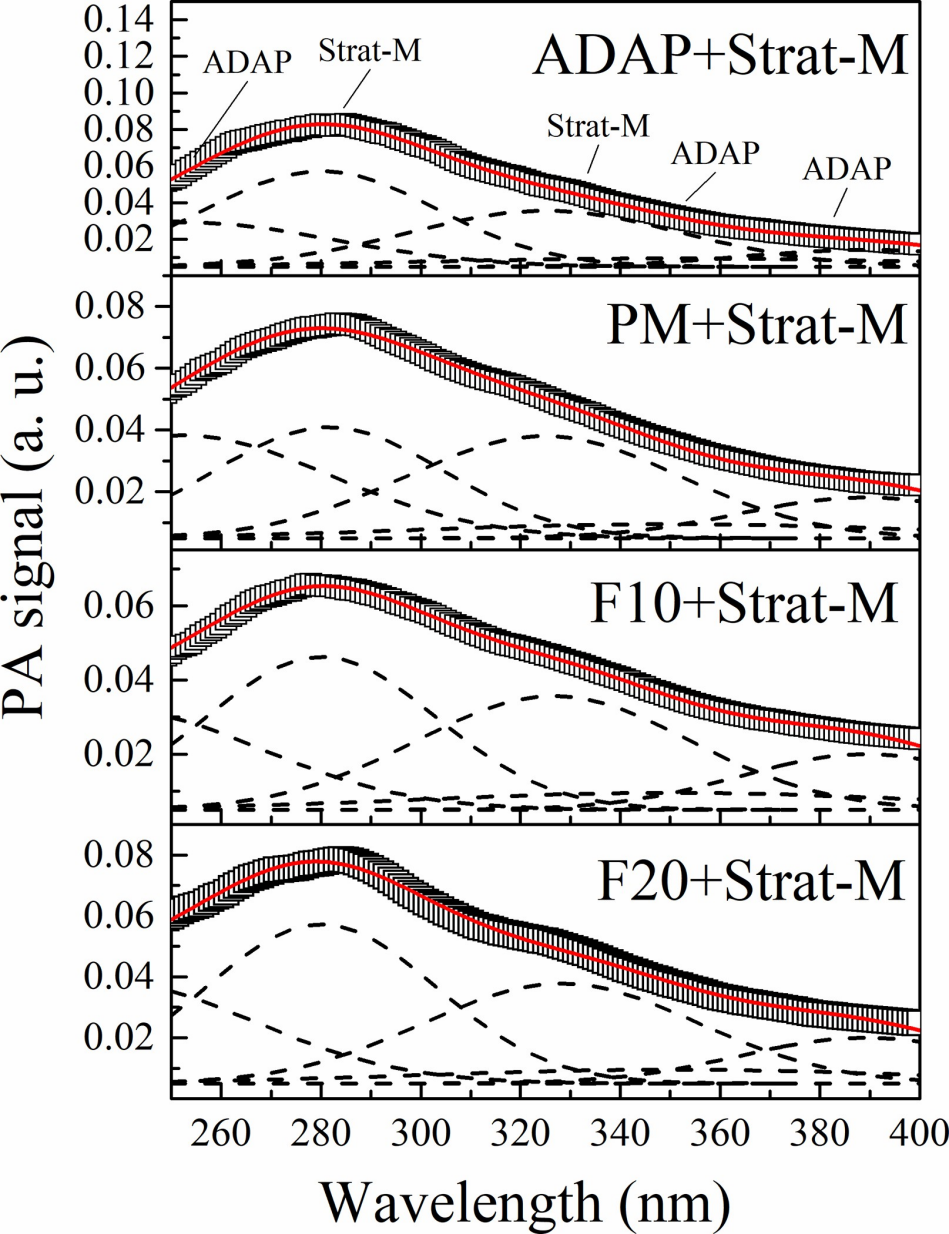
Photoacoustic spectra for the membranes after 15 min of application formulations (Formulation+Strat-M). Excitation in the internal side of the membrane with a 203 Hz modulation frequency (33 μm depth). The figure indicates the position of the peaks attributed to ADAP (250–269, 336 and 370–380 nm) and Strat-M (280, 300–350 nm).

**Fig 10:**
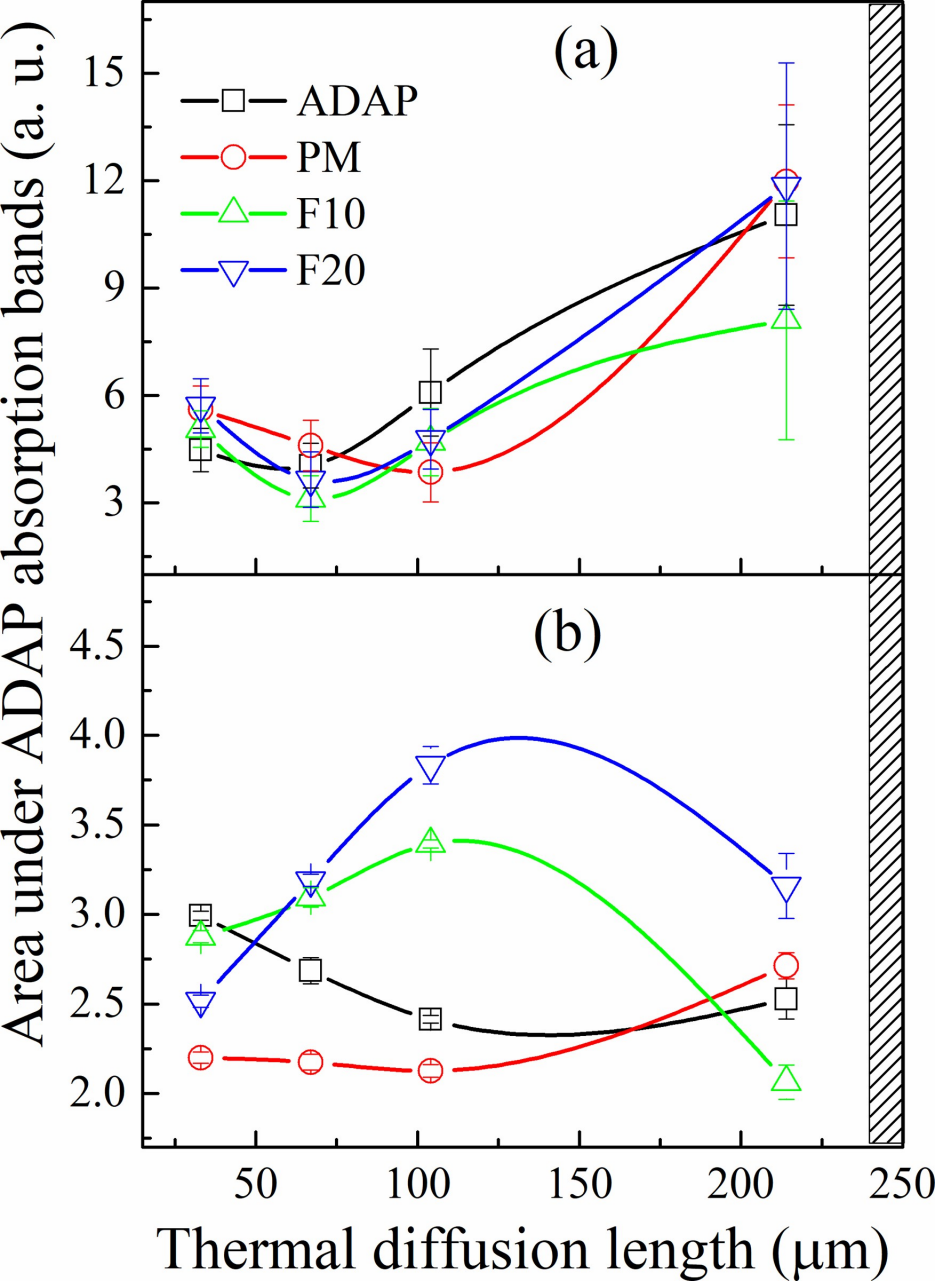
Evolution of ADAP permeation in the membrane as a function of thermal diffusion length after 15 min (a) and 3 h (b) of application obtained by the sum of the areas under the Gaussian curves related to ADAP (250–269, 336, and 370–380 nm). The hatched marks are approximately the positions where the formulations were applied. After 15 min, the highest drug concentration remains at approximately 210 μm for all formulations. After 3h, for ADAP and PM formulation, the drug permeates through all layers of the membrane until 33 μm. The F10 and F20 concentrate ADAP in 100 μm of penetration depth.

Fig 9 presents the results for excitation in the internal side of the membrane for a 203 Hz modulation frequency (33 μm depth) after 15 min of applying the formulations. The Gaussian fitting was performed based on five peaks centered at 280 and 300–350 nm from the membrane and 272, 336, and 369 from the formulations. The *in vitro* permeation of each formulation can be evaluated by summing the areas under the Gaussian curves related to ADAP for each thermal diffusion length. For this, the Gaussian fit was made for all the obtained spectra at each frequency (see supplementary material for details). The same procedure was performed for the membranes submitted to the formulations after 3 h of application. The results permeation as a function of the thermal diffusion length is presented in Fig 10. The figure shows that the peak centered at 272 nm (related to ADAP) shifted to approximately 250 nm. This shift can be considered for the adjust because it recurrs for the same frequencies and is in accordance with the literature [41,42].

Fig 10A reveals that after 15 min, the drug absorption was constant of the surface at 100 μm and was the most intense for 210 μm. Thus, the highest drug concentration remains at approximately 210 μm for all formulations. However, after 3 h, (Fig 10B) the behavior is different for all formulations. For the ADAP sample, the most intense absorption occurred close to the surface, indicating that the drug was detected and permeated through all layers of the membrane until 33 μm. Furthermore, most of the drug permeated through the membrane because the drug's contribution to larger diffusion lengths is lower. For the PM sample, the drug band absorption remains constant from 100 to 33 μm, indicating that the drug is spread through the membrane. The intensity of absorption of F10 presents a large increase of 210 to 100 μm and is slightly attenuated at 33 μm. Thus, a considerable amount of the drug permeates at 100 μm. The behavior of F20 is similar of the F10. However, the increase of intensity from 210 to 100 μm is much smaller, and the attenuation at the surface is most intense. This result shows that the encapsulation process controls the permeation of ADAP, demonstrating that ADAP-loaded microparticles are suitable for the pharmacological application.

According to the literature, it is possible to compare Strat-M membrane layers to human skin layers [39]. The poly(ether sulfone) compact surface is equivalent to the *stratum corneum* and the polyolefin region is equivalent to the dermis in the skin reaching the follicular pathway. Analyzing the Strat-M membrane from the internal side, our results provided an intensification of this equivalence. The equivalent *stratum corneum* region has a 210 μm penetration depth, and the *dermis* region has a 100 μm penetration depth.

Considering this equivalence, pure ADAP and PM spread through all layers from the *stratum corneum* to the *dermis*. In contrast, formulations F10 and F20 were concentrated in the 100 μm region, and the amount of the drug that diffused to the region equivalent to the subcutaneous tissue of the human skin is much lower when compared to the amount that diffused through for pure ADAP.

Summarizing these remarks, the ADAP-loaded PCL microparticles provide a suitable system for topical treatment. The encapsulation process considerably reduces the permeation of the drug into different membrane layers, indicating that the ADAP will target the *dermis* in future *in vivo* studies, which is the targeting region for acne treatment.

## Conclusion

ADAP-loaded PCL microparticles were effectively prepared by a simple emulsion/solvent evaporation method. Micrometer-sized formulations with high drug-loading efficiencies were obtained. No changes in FTIR assignments were recorded after the microencapsulation procedure. In addition, the photoacoustic spectroscopy showed that the microencapsulation decreased the *in vitro* transmembrane diffusion of ADAP, highlighting the particular characteristic of this technique to estimate the permeation of drugs through synthetic membranes. In summary, these formulations can be used in further innovative skin products intended for treating acne.

## Supporting information

S1 Fig Scheme of the raw data treatment obtained by photoacoustic spectroscopy for the membrane Strat M using 23 Hz as the modulation frequency. (DOCX)

S2 Fig Scheme of the raw data treatment obtained by photoacoustic spectroscopy from ADAP and formulations of ADAP-loaded PCL microparticles. (DOCX)

S3 Fig Scheme of the raw data treatment obtained by photoacoustic spectroscopy from the synthetic membrane treated with ADAP and formulations of ADAP-loaded PCL microparticles after 15 min and 3h of treatment. (DOCX)

S1 Table Adjust parameters obtained from Gaussian fitting performed on photoacoustic spectroscopy spectra for the membrane Strat M. (DOCX)

S2 Table Adjust parameters obtained from Gaussian fitting performed on photoacoustic spectroscopy spectra for ADAP and formulations. (DOCX)

S3 Table Adjust parameters obtained from Gaussian fitting for synthetic membrane after 15 min and 3h of treatment of ADAP and formulations. (DOCX)
