# Supplementary material for: Adapalene-loaded poly(ε-caprolactone) microparticles: Physicochemical characterization and in vitro penetration by photoacoustic spectroscopy
Source: PLoS One. 2019 Mar 21;14(3):e0213625. doi: 10.1371/journal.pone.0213625 (PMC6428289; doi:10.1371/journal.pone.0213625)
Supplement: S1 Fig — (DOCX) [file pone.0213625.s001.docx]

**Treatment of raw data obtained by photoacoustic spectroscopy**

S1 Fig. represents the scheme of the raw data treatment obtained by photoacoustic spectroscopy for the membrane Strat M.

The OPC technique and FEG-SEM results afforded information about the layers of the synthetic membrane. Before we used photoacoustic spectroscopy to study the membrane, it was necessary to calculate the modulation frequencies that could provide a depth profile through the membrane. We needed frequency values that were not multiples of the electrical power grid (60 Hz), which can cause noise effects. The pre-determined frequencies were 5, 23, 51, and 203 Hz, and these frequencies provided a depth scan of 210, 100, 67, and 33 µm, respectively, through the membrane.

Once the frequency modulation is determined, performing the photoacoustic spectroscopy for black carbon is necessary. This data provides the spectrum of the excitation from our setup composed of the Xenon Lamp, monochromator, filters, and lens. The next step was to obtain the raw data of the membrane Strat M, and for each frequency, we obtained one raw data. Each result was normalized by the black carbon spectrum.

*
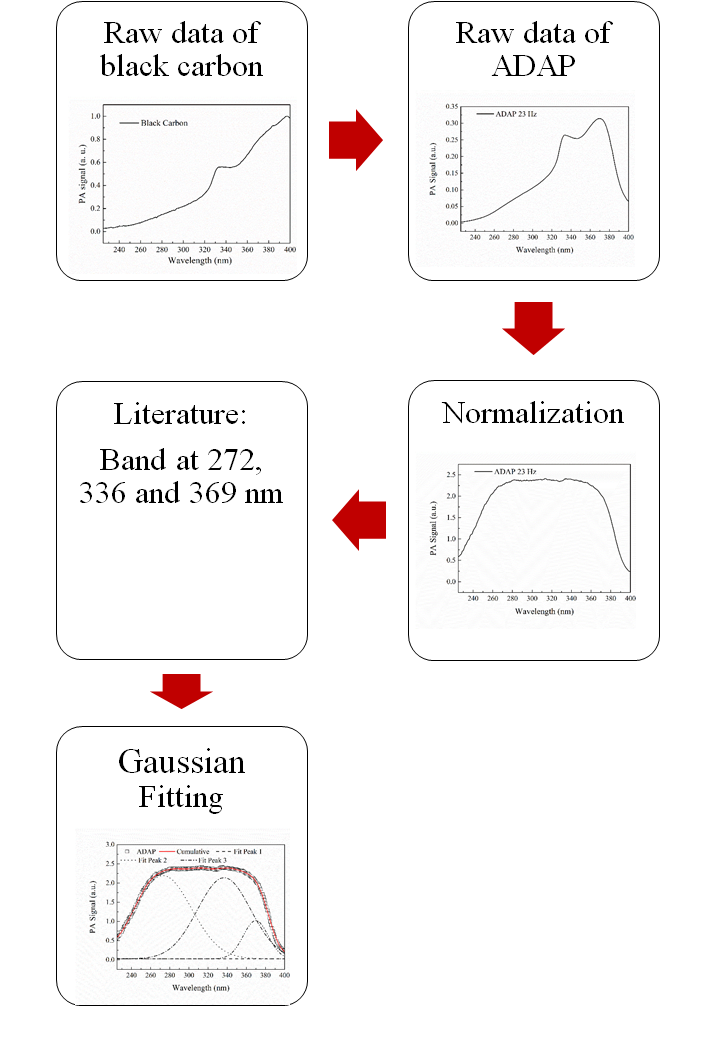
*

**S1 Fig.** Scheme of the raw data treatment obtained by photoacoustic spectroscopy for the membrane Strat M using 23 Hz as the modulation frequency.

According to literature, the synthetic membrane is composed of different types of polymers, such as poly(ether sulfone) and polyolefin. These polymers present absorptions in 280 and 300–350 nm. A Gaussian fitting was performed for each frequency for the membrane. The center of the 280 nm peak was maintained fixed. The adjust parameters of these fittings are presented in S1 Table.
