# Supplementary material for: Adapalene-loaded poly(ε-caprolactone) microparticles: Physicochemical characterization and in vitro penetration by photoacoustic spectroscopy
Source: PLoS One. 2019 Mar 21;14(3):e0213625. doi: 10.1371/journal.pone.0213625 (PMC6428289; doi:10.1371/journal.pone.0213625)
Supplement: S2 Fig — (DOCX) [file pone.0213625.s002.docx]

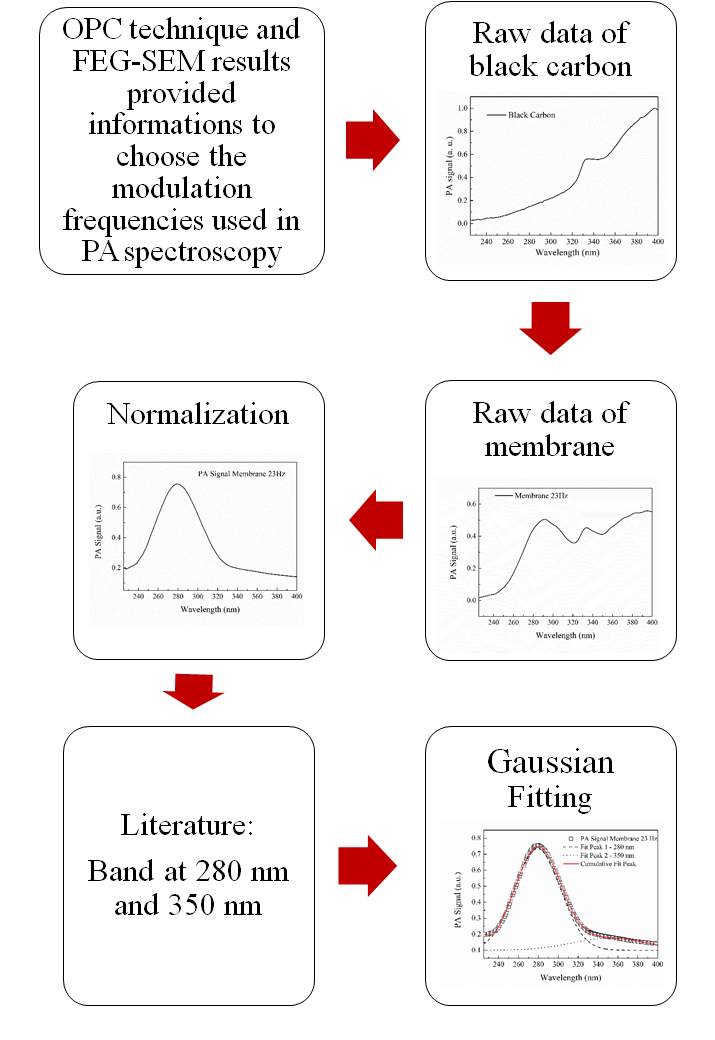


**S2 Fig.** Scheme of the raw data treatment obtained by photoacoustic spectroscopy from ADAP and formulations of ADAP-loaded PCL microparticles.
