# Supplementary material for: Adapalene-loaded poly(ε-caprolactone) microparticles: Physicochemical characterization and in vitro penetration by photoacoustic spectroscopy
Source: PLoS One. 2019 Mar 21;14(3):e0213625. doi: 10.1371/journal.pone.0213625 (PMC6428289; doi:10.1371/journal.pone.0213625)
Supplement: S1 Table — (DOCX) [file pone.0213625.s004.docx]

**S1 Table.** Adjust parameters obtained from Gaussian fitting performed on photoacoustic spectroscopy spectra for the membrane Strat M.

| Frequency (Hz)/ Depth (µm) | Fit Peak 1 (280 nm) | | | Fit Peak 2 (300–350 nm) | | |
| --- | --- | --- | --- | --- | --- | --- |
|  | Center (nm) | Width  (nm) | Area (a.u.) | Center (nm) | Width  (nm) | Area (a.u.) |
| 203/ 33 | 280 | 46 | 3.01 | 350 | 100 | 0.57 |
| 51/ 67 | 280 | 46 | 2.93 | 350 | 100 | 0.51 |
| 23/ 100 | 280 | 46 | 3.66 | 350 | 100 | 0.71 |
| 5 / 210 | 280 | 46 | 3.73 | 300 | 100 | 3.34 |

The next step was to analyze the absorption spectra of adapalene (ADAP) and formulations of ADAP-loaded PCL microparticles. The scheme of the raw data treatment obtained by photoacoustic spectroscopy for these samples is presented in S2 Fig. Since these samples can be treated as bulk, we used only a frequency modulation of 23 Hz. The other frequencies provided similar results. The raw data were normalized from the black carbon spectrum. From the literature, ADAP present absorption bands in 272, 336, and 369 nm, in this case, we performed a Gaussian fitting with three peaks while maintaining the center of each peak fixed at these absorptions. The results for ADAP and the formulations are presented in S2 Table.
