# Supplementary material for: Adapalene-loaded poly(ε-caprolactone) microparticles: Physicochemical characterization and in vitro penetration by photoacoustic spectroscopy
Source: PLoS One. 2019 Mar 21;14(3):e0213625. doi: 10.1371/journal.pone.0213625 (PMC6428289; doi:10.1371/journal.pone.0213625)
Supplement: S2 Table — (DOCX) [file pone.0213625.s005.docx]

**S2 Table.** Adjust parameters obtained from Gaussian fitting performed on photoacoustic spectroscopy spectra for ADAP and formulations

| Sample | Fit Peak 1 (272 nm) | | | Fit Peak 2 (336 nm) | | | Fit Peak 3 (369 nm) | | |
| --- | --- | --- | --- | --- | --- | --- | --- | --- | --- |
|  | Center (nm) | Width  (nm) | Area (a.u.) | Center (nm) | Width  (nm) | Area (a.u.) | Center (nm) | Width  (nm) | Area (a.u.) |
| ADAP | 272 | 59 | 67 | 336 | 55 | 60 | 369 | 25 | 14 |
| F10 | 272 | 59 | 67 | 332 | 52 | 55 | 365 | 25 | 12 |
| F20 | 272 | 59 | 69 | 334 | 55 | 54 | 365 | 25 | 11 |

The results presented in S2 Table were used in the data obtained for synthetic membrane submitted to the application of formulations. The scheme for this step is presented in S3 Fig. Each time treatment and modulation frequency was determined for each membrane, meaning that a new sample produced new raw data. Each raw data was normalized by black carbon. After the normalization, the Gaussian fitting was performed based on five peaks centered at 280 and 300–350 nm from the membrane and 272, 336, and 369 from the formulations. The adjust parameters are presented in S3 Table. The results show that peak1 shifts from the previously obtained 272 nm center to 252 nm. The band centered at 272 nm has a width of 60 nm and is in accordance with the literature. Besides, this shift is the same for each frequency and each depth, consequently we considered this peak to be from ADAP [see ref. 41 in the manuscript].

Finally, to obtain the evolution of ADAP permeation in the membrane, we considered the membrane as a control and summed the areas under the Gaussian curves related to ADAP (Peak 1, Peak 4, and Peak 5), and the results are presented in Fig. 9 in the manuscript. The values are presented in S3 Table (area total from ADAP).
