# Supplementary material for: Adapalene-loaded poly(ε-caprolactone) microparticles: Physicochemical characterization and in vitro penetration by photoacoustic spectroscopy
Source: PLoS One. 2019 Mar 21;14(3):e0213625. doi: 10.1371/journal.pone.0213625 (PMC6428289; doi:10.1371/journal.pone.0213625)
Supplement: S3 Table — (DOCX) [file pone.0213625.s006.docx]

**S3 Table**. Adjust parameters obtained from Gaussian fitting for synthetic membrane after 15 min and 3h of treatment of ADAP and formulations.

| Time of Treatment | Sample | Freq.  Hz | Depth  (µm) | Fit Peak 1 (250–270 nm)^a^ | | | Fit Peak 2 (280 nm)^b^ | | | Fit Peak 3  (300–350 nm)^b^ | | | Fit Peak 4 (336 nm)^a^ | | | Fit Peak 5 (369 nm)^a^ | | | Area total from ADAP |
| --- | --- | --- | --- | --- | --- | --- | --- | --- | --- | --- | --- | --- | --- | --- | --- | --- | --- | --- | --- |
|  |  |  |  | Center (nm) | Width  (nm) | Area (a.u.) | Center (nm) | Width  (nm) | Area (a.u.) | Center (nm) | Width  (nm) | Area (a.u.) | Center (nm) | Width  (nm) | Area (a.u.) | Center (nm) | Width  (nm) | Area (a.u.) | Area (a.u.) |
| 15 min | ADAP | 203 | 33 | 252 | 59 | 1.80 | 280 | 46 | 3.01 | 350 | 100 | 0.57 | 324 | 55 | 2.11 | 389 | 50 | 0.57 | 4.47 |
|  |  | 51 | 67 | 258 | 60 | 1.74 | 280 | 46 | 2.93 | 350 | 100 | 0.51 | 330 | 55 | 1.96 | 389 | 30 | 0.34 | 4.04 |
|  |  | 23 | 104 | 251 | 59 | 3.16 | 280 | 46 | 3.66 | 350 | 100 | 0.71 | 337 | 55 | 2.15 | 389 | 30 | 0.77 | 6.08 |
|  |  | 5 | 214 | 265 | 59 | 7.00 | 280 | 46 | 3.73 | 300 | 100 | 3.34 | 331 | 55 | 3.14 | 390 | 30 | 0.90 | 11.04 |
|  | PM | 203 | 33 | 252 | 59 | 2.50 | 280 | 46 | 3.01 | 350 | 100 | 0.57 | 325 | 55 | 2.28 | 389 | 50 | 0.83 | 5.61 |
|  |  | 51 | 67 | 258 | 60 | 2.06 | 280 | 46 | 2.93 | 350 | 100 | 0.51 | 330 | 55 | 2.07 | 388 | 30 | 0.48 | 4.60 |
|  |  | 23 | 104 | 251 | 59 | 2.19 | 280 | 46 | 3.66 | 350 | 100 | 0.71 | 333 | 55 | 1.38 | 389 | 30 | 0.28 | 3.86 |
|  |  | 5 | 214 | 265 | 59 | 7.67 | 280 | 46 | 3.73 | 300 | 100 | 3.34 | 337 | 55 | 3.37 | 390 | 30 | 0.93 | 11.98 |
|  | F10 | 203 | 33 | 252 | 59 | 2.02 | 280 | 46 | 3.01 | 350 | 100 | 0.57 | 326 | 55 | 2.11 | 389 | 50 | 0.93 | 5.06 |
|  |  | 51 | 67 | 258 | 60 | 1.64 | 280 | 46 | 2.93 | 350 | 100 | 0.51 | 330 | 55 | 1.24 | 389 | 32 | 0.24 | 3.12 |
|  |  | 23 | 104 | 251 | 59 | 2.36 | 280 | 46 | 3.66 | 350 | 100 | 0.71 | 334 | 55 | 1.78 | 389 | 30 | 0.56 | 4.70 |
|  |  | 5 | 214 | 265 | 59 | 5.45 | 280 | 46 | 3.73 | 300 | 100 | 3.34 | 343 | 55 | 2.10 | 389 | 30 | 0.55 | 8.10 |
|  | F20 | 203 | 33 | 252 | 59 | 2.51 | 280 | 46 | 3.01 | 350 | 100 | 0.57 | 328 | 55 | 2.26 | 389 | 50 | 0.94 | 5.71 |
|  |  | 51 | 67 | 258 | 60 | 1.99 | 280 | 46 | 2.93 | 350 | 100 | 0.51 | 334 | 55 | 1.29 | 389 | 30 | 0.37 | 3.65 |
|  |  | 23 | 104 | 251 | 59 | 2.37 | 280 | 46 | 3.66 | 350 | 100 | 0.71 | 330 | 55 | 1.88 | 389 | 30 | 0.53 | 4.78 |
|  |  | 5 | 214 | 265 | 59 | 6.42 | 280 | 46 | 3.73 | 300 | 100 | 3.34 | 323 | 55 | 4.15 | 388 | 30 | 1.28 | 11.85 |
| 3 h | ADAP | 203 | 33 | 252 | 59 | 2.38 | 280 | 46 | 3.01 | 350 | 100 | 0.57 | 327 | 55 | 1.88 | 389 | 50 | 1.11 | 5.37 |
|  |  | 51 | 67 | 258 | 60 | 2.83 | 280 | 46 | 2.93 | 350 | 100 | 0.51 | 339 | 55 | 1.49 | 389 | 50 | 1.19 | 5.51 |
|  |  | 23 | 104 | 251 | 59 | 3.12 | 280 | 46 | 3.66 | 350 | 100 | 0.71 | 338 | 55 | 1.20 | 389 | 50 | 1.21 | 5.53 |
|  |  | 5 | 214 | 265 | 59 | 3.78 | 280 | 46 | 3.73 | 300 | 100 | 3.34 | 340 | 55 | 1.49 | 390 | 50 | 1.03 | 6.30 |
|  | PM | 203 | 33 | 252 | 59 | 1.80 | 280 | 46 | 3.01 | 350 | 100 | 0.57 | 339 | 55 | 1.18 | 389 | 40 | 0.71 | 3.69 |
|  |  | 51 | 67 | 258 | 60 | 1.74 | 280 | 46 | 2.93 | 350 | 100 | 0.51 | 329 | 55 | 1.61 | 388 | 50 | 0.56 | 3.92 |
|  |  | 23 | 104 | 251 | 59 | 2.97 | 280 | 46 | 3.66 | 350 | 100 | 0.71 | 333 | 55 | 0.98 | 389 | 50 | 1.14 | 5.09 |
|  |  | 5 | 214 | 265 | 59 | 5.14 | 280 | 46 | 3.73 | 300 | 100 | 3.34 | 321 | 55 | 1.68 | 390 | 50 | 1.03 | 7.85 |
|  | F10 | 203 | 33 | 252 | 59 | 2.81 | 280 | 46 | 3.01 | 350 | 100 | 0.57 | 334 | 55 | 1.64 | 389 | 50 | 1.23 | 5.68 |
|  |  | 51 | 67 | 258 | 60 | 2.72 | 280 | 46 | 2.93 | 350 | 100 | 0.51 | 338 | 55 | 1.70 | 389 | 50 | 1.39 | 5.81 |
|  |  | 23 | 104 | 251 | 59 | 3.41 | 280 | 46 | 3.66 | 350 | 100 | 0.71 | 337 | 55 | 1.80 | 389 | 50 | 1.59 | 6.81 |
|  |  | 5 | 214 | 265 | 59 | 5.21 | 280 | 46 | 3.73 | 300 | 100 | 3.34 | 346 | 55 | 1.10 | 389 | 50 | 0.97 | 7.27 |
|  | F20 | 203 | 33 | 252 | 59 | 1.63 | 280 | 46 | 3.01 | 350 | 100 | 0.57 | 336 | 55 | 1.58 | 389 | 50 | 0.94 | 4.15 |
|  |  | 51 | 67 | 258 | 60 | 2.42 | 280 | 46 | 2.93 | 350 | 100 | 0.51 | 332 | 55 | 1.96 | 389 | 50 | 1.23 | 5.61 |
|  |  | 23 | 104 | 251 | 59 | 3.74 | 280 | 46 | 3.66 | 350 | 100 | 0.71 | 332 | 55 | 3.30 | 389 | 30 | 0.53 | 7.57 |
|  |  | 5 | 214 | 265 | 59 | 3.48 | 280 | 46 | 3.73 | 300 | 100 | 3.34 | 327 | 55 | 1.89 | 388 | 50 | 1.27 | 6.63 |

ª peak related to ADAP and ^b^ related to the membrane.
